# Childbirth fear and related factors among pregnant and postpartum women in Malawi

**DOI:** 10.1186/s12884-018-2023-7

**Published:** 2018-10-03

**Authors:** Madalitso Khwepeya, Gabrielle T Lee, Su-Ru Chen, Shu-Yu Kuo

**Affiliations:** 1 School of Nursing, College of Nursing, Taipei Medical University, Taipei, Taiwan; 2 Maternity Department, Machinga District Hospital, Liwonde, Malawi; 3 Applied Psychology, Faculty of Education, Western University, London, ON Canada; 4 School of Nursing, College of Nursing, Taipei Medical University, 250 Wuxing Street, Taipei, 11031 Taiwan

**Keywords:** Childbirth, Fear, Demographics, Obstetrics, Social support, Pregnancy, Postpartum period

## Abstract

**Background:**

Childbirth fear is a health concern in women living in high-income countries; however, little is known about childbirth fear among women living in low-income countries like Malawi. In this study, we explored childbirth fear and associated factors among pregnant and postpartum women in Malawi.

**Methods:**

A cross-sectional study of 152 pregnant and 153 postpartum women was conducted at a district hospital in Malawi. Participants were assessed for childbirth fear using the Wijma Delivery Expectancy/Experience Questionnaire (WDEQ). Demographic and obstetric variables were collected using a structured questionnaire. The Multidimensional Scale of Perceived Social Support (MSPSS) was used to measure social support. Using a multinomial logistic regression, factors related to childbirth fears were examined, namely demographic and obstetric characteristics, and social support.

**Results:**

The mean age of participants was 26 (standard deviation: 6.4) years. During pregnancy, 39% women reported a low level of fear, 41% reported moderate fear, and 20% reported high fear; while after birth, 49, 41, and 10% women reported low, moderate, and high fear, respectively. Pregnant women who were illiterate (odds ratio (OR): 5.0, *p* < 0.01) or unemployed (OR: 12.6, *p* < 0.01) were more likely to report moderate and high fear. Postpartum mothers who were illiterate (OR: 4.2, *p* < 0.01) or unemployed (OR: 11.8, *p* < 0.01) were more likely to have moderate and high fear. Furthermore, postpartum women who sustained perineal tears had significantly higher odds of experiencing moderate (OR: 5.3, *p* < 0.01) or high (OR: 19.9, *p* < 0.01) fear than their counterparts.

**Conclusions:**

Childbirth fear is common in Malawi, and pregnant women are more likely to experience high levels of fear than postpartum women. This study highlighted the connection between childbirth fear with mother’s education, employment, and perineal tears during delivery. Identifying and developing interventions for women with these associated characteristics is of clinical importance for the reduction of childbirth fear before and after childbirth in Malawi.

## Background

Childbirth fear is a health concern for women and their caregivers when expectant mothers are approaching birth or are in their postpartum transition [1, 2]. During pregnancy, women with childbirth fear often experience emotional distress [3, 4], and some might even request a cesarean section (CS) because of the fear [5, 6]. The CS rate has increased worldwide in recent years, and this may be associated with elective cesarean delivery on maternal request [7, 8]. After delivery, mothers with intense childbirth fear are more likely to have difficulties with maternal adjustment [9] and mother-child adaptation [10]. Previous research suggests that early identification of women at risk of childbirth fear and initiation of appropriate interventions are essential for women’s emotional well-being before and after birth [11]. However, most studies were conducted in high-income countries, and little is known regarding childbirth fear in low-income countries, such as Malawi.

Childbirth fear can be described as feelings of uncertainty and anxiousness before, during, or after the delivery [12, 13]. Childbirth fear in pregnant women is assessed based on their expectations of anticipatory delivery, while childbirth fear in postpartum women is measured with women’s actual childbirth experiences [12, 13]. Evidence suggests that socio-cultural factors and health care services are likely to influence the extent of childbirth fear and the consequences associated with the fear [14, 15]. In Malawi, maternal healthcare remains a major challenge due to high birth rates (41/1000 people) [16] and the lack of resources in the health care system [17–19]. Thus, mothers’ childbirth fear is often not assessed and cared for before and after the delivery. Despite considerable efforts are made to improve antenatal care (pregnant mothers have to attend at least 4 visits before delivery), hospital delivery with skilled birth attendant (i.e., by physicians or midwives), and postnatal coverage (including 1 week and 6 weeks of postpartum check-up) [18, 20, 21], mothers in Malawi are likely to be afraid of childbirth due to socio-cultural beliefs (i.e., witchcraft), health care providers’ attitudes (i.e., being rude), and lack of support/companion [22]. Childbirth fear continues to have a potential impact on women’s wellbeing, and this needs to be addressed directly with constant assessments and adequate care.

Childbirth fear is common in both pregnant and postpartum women in developed countries. The prevalence of childbirth fear is estimated to range from 16 to 27% during pregnancy [5, 11–13], and around 22% after birth [13]. A study in Sweden by Alehagen et al. [23] found a positive association between childbirth fear during pregnancy and postpartum period. Zar et al. reported that women who were more afraid of childbirth during pregnancy were more likely to have fears during and after labor [24]. A prospective correlational design study by Fenwick et al. found that antenatal fear significantly predicted the levels of postnatal fear [25]. A review of the existing literature suggests that most of the studies on childbirth fear have either focused on pregnant or postpartum mothers, but not on both [23]. Understanding childbirth fear in both pregnant and postpartum mothers is necessary for developing comprehensive psychological maternal care programs.

Previous studies on childbirth fear suggest that the demographic, obstetric characteristics, and social support, are potential factors associated with childbirth fear among childbearing women [26–28]. Younger women are more likely to experience elevated fears than older women during childbirth [26]. Women who are not educated or not working are more likely to be fearful than their counterparts [26, 28]. First-time mothers are more prone to have childbirth fear than parous women [11, 29]. Mothers who sustain perineal tears during delivery are at increased risk of being fearful [27]. A lack of social support is associated with childbirth fear in women [26]. To this date, no attempt has been made to understand such factors in Malawian women. Therefore, exploring these factors affecting women’s childbirth fear is the first step towards establishing regular assessments, proper educational interventions, and the provision of mental health care to childbearing women in Malawi.

Given that previous research indicated that women’s childbirth fear is related to their overall health and emotional well-being, it is imperative to explore such phenomena in Malawian women. To fill in gaps in the literature, we conducted a cross-sectional study to explore factors associated with childbirth fear in pregnant and postpartum women in Malawi. The specific aims of this research were to: (1) investigate the proportion of women who experience different levels of childbirth fear, and (2) examine factors such as demographic and obstetric characteristics, and social support, associated with childbirth fear in pregnant and postpartum women.

## Methods

### Study design

A cross-sectional study was conducted from August to September, 2015 at a district hospital in Malawi. The study site was one of the major referral heath facilities in Malawi with more than 22 health centers referring their patients for medical care. The hospital provided services to about 538,345 people and registered at least 1500 deliveries a month [30].

### Study participants

Participants in this study were pregnant and postpartum women receiving prenatal and postnatal care at this district hospital. Potential participants were approached by the researchers or were referred by the nurse-midwives to participate in the study after receiving their antenatal and postpartum care. We included pregnant and postpartum women who a) were aged ≥18 years; b) understood and spoke Chichewa; c) were in their second or third trimester with a singleton pregnancy, or d) had delivered their babies less than 6 weeks previously of the postpartum period. Women who had high-risk perinatal conditions such as preeclampsia/eclampsia, hemorrhaging, or a medical or mental illness were excluded. This was confirmed by checking on patient-held health records and medical records. Considering this is the first attempt exploring childbirth fear in Malawi, both normal vaginal delivery and cesarean section women were included. Using G*Power calculator, the sample size calculation of the study was based on previous studies by Ryding et al. [31] and Johnson et al. [32]. To obtain a power of 80% at 5% significance level with an effect size of 0.4 in a two-tailed independent *t*-test, a sample of 200 participants with 100 in each group was required.

The study protocol was approved by the Institutional Review Board of the National Health Science Research Committee (NHSRC), Ministry of Health, Malawi. Furthermore, an approval was obtained from the study setting to conduct the study. Eligible participants provided written informed consent prior to their participation. An informed consent form was given to each participant to read and fill out. If the participants were illiterate, the researchers read out the consent form and helped the participants to fill out the questionnaires.

### Measures

#### Translation process

The Wijma Delivery Expectancy/Experience Questionnaire (W-DEQ) A (expectancy) and B (experience) [13] and Multidimensional Scale of Perceived Social Support (MSPSS) [33, 34] were translated into Chichewa language following recommended guidelines by Wild et al. [35]. First, we obtained the permission to use the instruments from the original authors. Second, the original English versions were translated into Chichewa by a bilingual translator (forward translation). The translated versions were then checked and discussed for wording and clarity by several Malawian-speaking experts, including the researcher who had backgrounds in midwifery (reconciliation). Third, the Chichewa versions were translated back into English by three independent bilingual translators (back translation). The original and back-translated questionnaires were discussed and compared for clarity and inconsistencies to reach a consensus on the final versions. Fourth, a pilot study was conducted to assess the wording and adequacy of translated versions. The participants in the pilot study were 15 pregnant and 15 postpartum women at the antenatal check-up and postpartum ward in Malawi.

#### Childbirth fear

Childbirth fear was measured using the Chichewa version of the Wijma Delivery Expectancy/Experience Questionnaire (WDEQ) versions A and B [13]. Pregnant women were assessed using version A, and postpartum women were assessed using version B. The WDEQ is a 33-item self-reported questionnaire with a 6-point Likert scale ranging from 0 (not at all) to 5 (extremely), with a total score from 0 to 165. A score of ≤37represents low fear, of 38~ 65 represents moderate fear, and of ≥66 represents high fear [13]. The original WDEQ reported a Cronbach’s alpha of > 0.87 in nulliparous and multiparous women [13]. Our pilot study had a Cronbach’s alpha of 0.78. The content validity index (CVI) was 0.93~ 0.95. In this study, the Cronbach’s alphas of the Chichewa version of the WDEQ ranged 0.77~ 0.79.

#### Demographic and obstetric characteristics

Demographic and obstetric variables were examined using a structured questionnaire for each participant. Furthermore, these were checked in the patient-held health records with participants’ permission. Demographic characteristics included age, educational level, employment status, and monthly income [26]. Obstetric characteristics included parity, mode of birth, perineal tears, and pregnancy complications [36].

#### Social support

Social support was assessed using the Multidimensional Scale of Perceived Social Support (MSPSS). The original questionnaire was developed by G. Zimet [34]. The MSPSS is a 12-item self-reported questionnaire with a 7-point Likert scale that ranges from 1 (very strongly disagree) to 7 (very strongly agree), with a total score ranging 12~ 84 [33]. The MSPSS measures the perception of social support from family, friends, and significant others, with higher scores indicating better perceived social support. The original MSPSS reported a Cronbach’s alpha of 0.88 [34]. Our pilot study had a Cronbach’s alpha of 0.83. The CVI was 1. In this study, the Cronbach’s alphas of the Chichewa MSPSS ranged 0.81~ 0.84.

### Statistical analysis

The Statistical Package for Social Sciences (SPSS) version 21 (SPSS, Chicago, IL, USA) was used for all analyses. Descriptive statistics summarizing participants’ characteristics included the percentage, frequency, mean, and standard deviation (SD). Of bivariate analyses, *t*-tests were used for continuous variables (age and childbirth fear total score), and χ^2^ tests (age, educational level, employment status, income, parity, preferred/mode of birth, perineal tears, past pregnancy complications, social support, and childbirth fear class) were used for categorical variables. Potential confounding variables were identified when the variables were significant at ≤0.10 and were then included for subsequent analysis. A multinomial logistic regression was used to determine factors associated with childbirth fear, and an α level of ≤0.05 was considered significant. Missing data ranged 0%~ 3.9% for all variables except age and are indicated with ^++^ in Table 1. The age variable had 9.2% missing values due to women’s high illiteracy levels. Women who did not know their birth date were coded as having a missing value.

**Table 1:** Characteristics of participants

Variable	Pregnant women (*N* = 152)		Postpartum women (*N* = 153)		χ^2^	*p*
	*n*	(%)	*n*	(%)		
Age (years)^++^, Mean (SD)	26.3	(6.6)	26.1	(6.3)		
< 25	73	(48.0)	75	(49.0)	0.01	0.91
≥ 25	65	(42.8)	65	(42.5)		
Education level^++^						
Illiterate	24	(15.8)	29	(19.0)	0.66	0.72
Primary school	99	(65.1)	94	(61.4)		
Secondary and higher	28	(18.4)	30	(19.6)		
Employment status^++^						
Unemployed	23	(15.1)	13	(8.5)	3.30	0.07
Employed	128	(84.2)	140	(91.5)		
Income per month						
< MK20,000	115	(75.7)	125	(81.7)	1.66	0.20
MK20,000~ 50,000	37	(24.3)	28	(18.3)		
Parity						
Nulliparous	30	(19.7)	43	(28.1)	2.93	0.09
Multiparous	122	(80.3)	110	(71.9)		
Preferred MOD^a^/Actual MOD^b++^						
Caesarean delivery	3	(2.0)	10	(6.5)	3.70	0.05
Normal vaginal birth	145	(95.4)	143	(93.5)		
Perineal tears^++^						
Yes	~	~	20	(13.1)	~	~
No			127	(83.0)		
Past pregnancy complications^++^						
Yes	33	(21.7)	44	(28.8)	2.10	0.15
No	119	(78.2)	108	(70.6)		
Social support, Mean (SD)	5.7	(0.9)	5.4	(0.9)		
Low/Moderate	35	(23.0)	47	(30.7)	2.30	0.13
High	117	(77.0)	106	(69.3)		

SD, Standard deviation; ^a^ Preferred Mode of Birth for pregnant women; ^b^ Actual Mode of Birth for postpartum women; *MK* Malawi Kwacha currency (US$1 ≈ Malawi Kwacha (MK)725); ^++^Total amounts do not either add up to *n* = 152 (100%) for pregnant women or *n* = 153 (100%) for postpartum women

## Results

### Participants

Of the 307 eligible women who were invited and agreed to participate in this study, two pregnant women were excluded due to high-risk conditions of antepartum hemorrhaging and preterm labor. In total, 305 women participated, including 152 pregnant and 153 postpartum women. The mean age of pregnant women was 26.3 with Standard Deviation (SD) of 6.6 (Table 1). Most pregnant women were young (< 25 years; 48%), had a primary school education (65%), were employed (84%), and earned less than Malawi Kwacha (MK) 20,000/month (76%). The majority of women were multiparous (80%) and preferred to have a normal vaginal birth (95%) rather than a CS. More than half reported no complications of past pregnancies (78%) and received high social support (77%). Our participants are representative of the population of childbearing women in Malawi. According to the Malawi Demographic Health Survey (MDHS) and other reports, the mean age of childbearing women is 28.3 years. About 62% of women aged 15–49 have primary education, and 70% of married women are employed [21, 37].

Characteristics of the postpartum women were similar to those of pregnant women. The mean age of postpartum women was 26.1 (SD:6.3) (Table 1). The majority of the postpartum women were young (< 25 years; 49%), had a primary school education (61%), were employed (92%), and earned less than MK20,000/month (82%). More than half were multiparous (72%), had had a normal vaginal birth (94%), and had sustained no perineal tears (83%). Most women indicated no complications with past pregnancies (71%) and received high social support (69%). The χ^2^ test showed that pregnant and postpartum women had similar characteristics (*p* = 0.07~ 0.91), except for the preferred mode of birth/actual mode of birth.

### Levels of childbirth fear in pregnant and postpartum women

Pregnant women reported higher levels of fear (mean, 47) compared to postpartum women (mean, 42) (Table 2). Of the 152 pregnant women, 59 (39%) reported low levels of fear, 62 (41%) were moderately fearful, and 31 (20%) were highly fearful. Of the 153 postpartum women, 75 (49%) reported low levels of fear, 63 (41%) were moderately fearful, and 15 (10%) were highly fearful. Overall, compared to the pregnant women, the postpartum women reported a higher proportion of low fear (χ^2^ = 7.48, *p* = 0.02).

**Table 2:** Childbirth fear in pregnant and postpartum women

Variable	Pregnant women (*N* = 152)		Postpartum women (*N* = 153)		Statistical value	*p*
	*n*	(%)	*n*	(%)		
Childbirth fear, Mean (SD)	46.9	(18.8)	42.2	(14.6)	2.48^a^	0.01
Low (≤37)	59	(38.8)	75	(49.0)	7.48^b^	0.02
Moderate (38~ 65)	62	(40.8)	63	(41.2)		
High (≥66)	31	(20.4)	15	(9.8)		

Note: *SD* Standard deviation; ^a^ *t*-test; ^b^ Chi-squared test

### Factors associated with childbirth fear

Tables 3 and 4 shows the demographic and obstetric characteristics, and social support factors for the three levels of childbirth fear in pregnant and postpartum women that were related to childbirth fear in the multinomial logistic regression using ‘Low fear’ (WDEQ score of ≤37) as the reference group.

**Table 3:** Relationships of baseline characteristics with childbirth fear in pregnant women (*N* = 152)

Variable	Low fear (*n* = 59)		Moderate fear (*n* = 62)					High fear (*n* = 39)				
	*n*	(%)	*n*	(%)^a^	*p*	aOR (95% CI)^b^	*p*	*n*	(%)^a^	*p*	aOR (95% CI)^b^	*p*
Age (years)												
< 25	31	(56.4)	26	(44.8)	0.22	0.7 (0.3~ 1.6)	0.36	16	(64.0)	0.52	1.0 (0.3~ 3.1)	0.93
≥ 25	24	(43.6)	32	(55.2)		1.00		9	(36.0)		1.00	
Parity												
Nulliparous	14	(23.7)	11	(17.7)	0.42	1.3 (0.4~ 4.3)	0.62	5	(16.1)	0.40	1.0 (0.2~ 4.9)	0.96
Multiparous	45	(76.3)	51	(82.3)		1.00		26	(83.9)		1.00	
Educational level												
Illiterate	6	(10.2)	15	(24.6)	0.01	5.0 (1.2~ 20.2)	0.03	3	(9.7)	0.44	1.8 (0.2~ 17.8)	0.61
Primary school	37	(62.7)	38	(62.3)	0.14	2.2 (0.8~ 6.1)	0.13	24	(77.4)	0.12	3.0 (0.7~ 13.1)	0.16
Secondary and above	16	(27.1)	8	(13.1)		1.00		4	(12.9)		1.00	
Employment status												
No	4	(6.8)	5	(8.2)	0.77	1.5 (0.4~ 6.3)	0.57	14	(45.2)	< 0.001	12.6 (3.1~ 50.7)	< 0.001
Yes	55	(93.2)	56	(91.8)		1.00		17	(54.8)		1.00	
Social support												
Low/Moderate	8	(13.6)	14	(22.6)	0.20	1.5 (0.6~ 4.1)	0.43	13	(41.9)	< 0.01	3.0 (0.9~ 10.2)	0.09
High	51	(86.4)	48	(77.4)		1.00		18	(58.1)		1.00	

^a^The significance level of a univariate odds ratio denoted in this column if significant. ^b^ Adjusted odds ratio (aOR) and its 95% confidence interval (CI), obtained after statistical adjustment for all of the variables listed in this table

**Table 4:** Relationships of baseline characteristics to childbirth fear in postpartum women (*N* = 153)

Variable	Low fear (*n* = 75)		Moderate fear (*n* = 63)					High fear (*n* = 15)				
	*n*	(%)	*n*	(%)^a^	*p*	aOR (95% CI)^b^	*p*	*n*	(%)^a^	*p*	aOR (95% CI)^b^	*p*
Age (years)												
< 25	36	(49.3)	31	(56.4)	0.43	1.0 (0.4~ 2.4)	0.99	8	(66.7)	0.27	1.8 (0.3~ 11.5)	0.52
≥ 25	37	(50.7)	24	(43.6)		1.00		4	(33.3)		1.00	
Parity												
Nulliparous	17	(22.7)	21	(33.3)	0.16	2.1 (0.8~ 5.9)	0.16	5	(33.3)	0.38	1.9 (0.3~ 14.2)	0.52
Multiparous	58	(77.3)	42	(66.7)		1.00		10	(66.7)		1.00	
Educational level												
Illiterate	9	(12.0)	17	(27.0)	0.21	4.2 (1.1~ 16.3)	0.04	3	(20.0)	0.63	0.3 (0.0~ 8.4)	0.47
Primary school	52	(69.3)	33	(52.4)	0.39	1.2 (0.4~ 3.3)	0.77	9	(60.0)	0.77	2.2 (0.3~ 17.0)	0.45
Secondary and above	14	(18.7)	13	(20.6)		1.00		3	(20.0)		1.00	
Employment status												
No	4	(5.3)	6	(9.5)	0.35	3.0 (0.6~ 14.6)	0.17	3	(20.0)	0.07	11.8 (1.3~ 108.4)	0.03
Yes	71	(94.7)	57	(90.5)		1.00		12	(80.0)		1.00	
Mode of birth												
Caesarean delivery	3	(4.0)	3	(4.8)	0.83	0.2 (0.0~ 2.4)	0.23	4	(26.7)	< 0.01	8.4 (0.8~ 86.7)	0.08
Normal vaginal birth	72	(96.0)	60	(95.2)		1.00		11	(73.3)		1.00	
Perineal tears												
Yes	5	(6.7)	9	(15.3)	0.12	5.3 (1.1~ 25.3)	0.04	6	(46.2)	< 0.01	19.9 (2.4~ 162.7)	< 0.01
No	70	(93.3)	50	(84.7)		1.00		7	(53.8)		1.00	
Social support												
Low/Moderate	23	(30.7)	17	(27.0)	0.64	0.8 (0.3~ 1.9)	0.61	7	(46.7)	0.24	1.7 (0.3~ 8.4)	0.53
High	52	(69.3)	46	(73.0)		1.00		8	(53.3)		1.00	

^a^The significance level of a univariate odds ratio denoted in this column if significant. ^b^ Adjusted odds ratio (aOR) and its 95% confidence interval (CI), obtained with statistical adjustment for all of the variables listed in this table

Results of the univariate analyses for pregnant women showed that being illiterate, not working, and receiving less social support were significantly associated with a higher level of fear, whereas age and parity were not (Table 3). After adjusting for age, parity, educational level, employment status, and social support as the variables listed in Table 3, the adjusted ORs (aORs) of all of the variables except for social support remained significant for moderate and high fear.

Results of the univariate analysis for postpartum women showed that having an emergency CS and sustaining perineal tears significantly increased the odds of having higher fear (Table 4). After adjusting for age, parity, mode of birth, and social support as listed in Table 4, being illiterate (aOR = 4.2) and having perineal tears (aOR = 5.3) were associated with moderate fear. Not working (aOR = 11.8) and sustaining perineal tears (aOR = 19.9) increased the risk of high fear.

## Discussion

The purpose of this study was to examine childbirth fear and related factors in pregnant and postpartum women in Malawi. Our results indicated that over 50% of women experienced moderate or high fear during the perinatal stage. In particular, pregnant women who were illiterate or unemployed were prone to have higher levels of childbirth fear. After birth, mothers who were illiterate, were unemployed, and had sustained perineal tears were at increased risk of having elevated fears. Perceived social support was not significantly associated with childbirth fear in pregnant or postpartum women. Our study extends the literature by examining factors correlated with women’s childbirth fear in Malawi, a developing country with a unique culture.

### Childbirth fear

Our study revealed that childbirth fear is common in Malawi. During pregnancy, about 20% of women had a relatively high level of fear (with a score on the WDEQ-A of ≥66), a distribution similar to previous studies that used the same assessment tool in Canadian (25%) [38], Australian (24%~ 27%) [25, 39], and Swedish (26%) [24] women. A recent review suggested that most studies on childbirth fear were conducted in high-income countries, and the prevalence estimate greatly varied due to different measures and definitions [40]. Results of our study indicated that childbirth fear also exists among women in a low-income country, and the level of childbirth fear was similar to those of women in high-income countries despite differences in birth rates, medical resources and practices, and cultural values [41, 42]. Childbirth as a natural event accompanied by labor pains and potentially unpredictable variables in the process might partly explain why women in different regions or cultures also share similar levels of childbirth fear [7, 43]. A study by Fenwick et al. revealed that women were concerned about the length of time experiencing pain and were more receptive to chemical forms of pain reduction [43]. However, women in a low-resource setting might not opt to receive pain medication during childbirth, as such medications are rare and expensive [44]. Offering women the option to receive pain medication and making the medication available upon request might ease women’s childbirth fears. In high-resource countries, mental health care is usually an integrated part of healthcare systems. Besides medically necessary resources, care of women’s emotional well-being during and after pregnancy often includes educational sessions on childbirth, opportunities for professional consultation, and meetings with various social support groups [45–47]. Addressing mental healthcare issues can be a challenging task in Malawi. Given the country’s high rates of childbirth [15], maternal mortality [48], and infant mortality [49], health care for women typically focuses on physical health while ignoring mental health. Our data clearly indicated that the level of childbirth fear was similar between Malawian women and women in high-resource countries, suggesting that the need for mental health care for women in Malawi is also essential to their overall well-being.

Our data indicated that relatively few postpartum women (10%) reported a high level of fear (WDEQ ≥66), compared to pregnant women. As reported by Fenwick et al. [25] and Zar et al. [12], women’s fear levels over the perinatal period decreased for nulliparous and multiparous women in the high-fear group. However, Fenwick et al. [25] also pointed out that women who experienced childbirth interventions (e.g., a CS) may have an increased level of postpartum fear, and therefore, special attention and care are necessary for this population. Our study found postpartum women had low childbirth fear and this might possibly be related to the fact that the majority of our study sample were multiparous and had had a vaginal birth without additional interventions. Although our data also indicated a low proportion of fear after delivery, women’s fear did not completely cease or disappear. Therefore, it is necessary to provide assessments and care on a regular basis following childbirth in order to prevent potential adverse outcomes that might have an impact on future pregnancies and childbirths [4].

### Demographic factors associated with childbirth fear

In this study, we found a significant association between childbirth fear and women’s educational level. Women who were illiterate were more likely to report a higher level of childbirth fear during pregnancy and in the postpartum period than their more highly educated counterparts. Previous work by Laursen et al. [26] and Salomonsson et al. [28] also found a significant association between these two factors, although the relationship between them was not clear. It is possible that uneducated women do not usually comprehend childbirth information and make informed choices. Conversely, Saisto et al. found no significant association between women’s educational backgrounds and their fear of childbirth [50]. More research is needed to examine the association between women’s educational level and childbirth fear. In any case, providing educational programs designed specifically for illiterate women in Malawi may help reduce their fears and promote their emotional well-being before and after childbirth.

We found that a woman’s unemployment status was significantly associated with a higher level of fear during pregnancy and after childbirth. Our results concurred with previous studies in Denmark [26] and Finland [50], which also suggested that unemployed women were at higher odds of having childbirth fear. Saisto et al. [50] suggested that unemployment was a socioeconomic factor that may be linked to women’s childbirth fear. On the contrary, other researchers reported that employed women were more likely to experience childbirth fear and seek treatment for psychological well-being [39, 51]. Toohill et al. [39] further reported that mothers who were employed were more likely to have a high level of fear, compared to mothers who were not engaged in paid work. Given the mixed results of women’s employment status with childbirth fear, it is important to conduct more studies to further understand such associations in Malawian women. Our results suggest a need to incorporate mental health care into perinatal care for unemployed women in Malawi.

### Obstetric factors associated with childbirth fear

The mode of birth was significantly related to childbirth fear in this study. However, the result was statistically insignificant after adjusting for other variables. Our results are similar to previous findings by Fenwick et al. [25] and Johnson et al. [32], who found no association between childbirth fear and a CS delivery. Johnson et al. in their study specified that most women were generally more frightened of childbirth (mean scores of the WDEQ of > 60) across all types of delivery [32]. However, Ryding et al. did not find the same association but found that women who underwent a CS were at a greater risk of anxiety, had poorer stress-coping abilities, and had higher levels of childbirth fear [52]. Childbirth fear being associated with a CS may be attributed to negative birth experiences, and some women considered such a birth intervention to be a traumatic event [53]. Additionally, a CS is major surgery usually accompanied by poor physical and psychological outcomes [54–56]. Medical systems and cultural factors of different countries might contribute to the mixed results of childbirth fear and CS delivery [25]. In our study, most mothers clearly indicated their preference for a vaginal birth as opposed to a CS. As the CS rate is rising globally (26%) [8], the rate in Malawi is only around 5% [57], possibly due to limited access to CSs in Malawi. Although current conditions in Malawi do not allow women to have a CS by choice, this might not necessarily have an adverse impact on their childbirth fear. Instead, this creates an opportunity for women to reflect on their childbirth experiences in relation to their fears under professional guidance as recommended by other studies [46] and not to choose a CS due to fear of childbirth. Future investigations on the association between a CS delivery and childbirth fear among perinatal women are needed.

We found that women with perineal damage (tears) after delivery tended to be at risk of childbirth fear. A study by Areskog et al. reported that women’s fears were associated with physical damage to themselves [58]. Serçekuş et al. also reported a similar result in their qualitative study that women’s fears may be associated with episiotomies [27]. A study conducted in Australia reported that concern about perineal tearing may lead to childbirth fear in childbearing women and the decision to have a CS as an easier option for birth [43]. In Malawi, episiotomy is not a standard practice. Therefore, the significant association between women’s fear and perineal tears might be related to pains experienced during and after suturing. Furthermore, potential complications that underline sexual activities resulting from perineal tears might be of concern for some women [59]. Concerns about perineal damage by Malawian women warrant the need for healthcare professionals to attend to women’s pain levels by administering anesthesia upon a woman’s request, provide instructions for perineal management, inform the patient about potential complications, and facilitate checkup visits [60]. Furthermore, it is important that healthcare professionals take cautious actions to minimize the extent of perineal damage during birth in order to optimize birth and emotional outcomes.

### Social support

Women in our study who received less social support during pregnancy reported higher levels of childbirth fear. However, the result was statistically insignificant after adjusting for other variables. Previous studies also reported that a woman’s lack of social support was associated with childbirth fear [7, 26, 61], as having social support may act as a buffer in stressful situations [62]. However, Haines et al. refuted such a relationship and found that personal or social characteristics of women had no connection with childbirth fear, especially in rural townships with a sense of community that reduced the need for direct support [63]. This potentially explains why the results were not significant after adjusting for other confounders in our study. In Africa including Malawi, reproductive health issues such as childbirth are considered a woman’s responsibility [64, 65]. Thus, women rely on each other in times of childbirth [66], hence fostering community support. Although social support was not significant after adjusting for other variables in pregnant women, identifying and providing long-lasting social support [50, 61] by families and significant others are vital during pregnancy.

### Study limitations

When interpreting results of this study, some limitations need to be considered. Our study used a cross-sectional design to explore possible factors associated with childbirth fear which cannot show the cause-and-effect relationships. However, as the first study investigating childbirth fear in Malawi, our findings provide a basis for understanding childbirth fear in pregnant and postpartum women. Future studies can consider study designs that are longitudinal in nature to draw such causal inferences. The generalizability of results to high-risk women and other hospitals is limited, because our study only included women with no complications in a single hospital. We recommend further studies to incorporate high-risk women and women in various districts and central hospitals for the purpose of increasing generalizability. As most women in our study were illiterate, the researchers helped the illiterate participants to fill out the questionnaires. This could potentially underestimate their responses if they perceived less privacy.

## Conclusions

Childbirth fear is common in Malawian women. Pregnant women tend to report high levels of childbirth fear, compared to postpartum women. The demographic and obstetric characteristics of the women were associated with childbirth fear during pregnancy and after childbirth. Early identification of women at risk of childbirth fear is of clinical importance in order to improve the health care for women during pregnancy and after the delivery in Malawi. Therefore, clinical attention to childbirth fear with comprehensive assessments and mental health care in Malawi is warranted.

## Abbreviations

MK: Malawi Kwacha
MSPSS: Multidimensional Scale of Perceived Social Support
NHSRC: National Health Science Research Committee
WDEQ: Wijma Delivery Expectancy/Experience Questionnaire
